# The Corepressor *Tle4* Is a Novel Regulator of Murine Hematopoiesis and Bone Development

**DOI:** 10.1371/journal.pone.0105557

**Published:** 2014-08-25

**Authors:** Justin C. Wheat, Daniela S. Krause, Thomas H. Shin, Xi Chen, Jianfeng Wang, Dacheng Ding, Rae’e Yamin, David A. Sweetser

**Affiliations:** 1 Department of Pediatrics, Divisions of Pediatric Hematology/Oncology and Medical Genetics, Massachusetts General Hospital, Boston, Massachusetts, United States of America; 2 Center for Regenerative Medicine and Cancer Center, Massachusetts General Hospital, Boston, Massachusetts, United States of America; 3 Department of Pathology, Massachusetts General Hospital, Boston, Massachusetts, United States of America; 4 Department of Molecular and Translational Medicine, Boston University School of Medicine, Boston, Massachusetts, United States of America; Rutgers - New Jersey Medical School, United States of America

## Abstract

Hematopoiesis is a complex process that relies on various cell types, signaling pathways, transcription factors and a specific niche. The integration of these various components is of critical importance to normal blood development, as deregulation of these may lead to bone marrow failure or malignancy. *Tle4*, a transcriptional corepressor, acts as a tumor suppressor gene in a subset of acute myeloid leukemia, yet little is known about its function in normal and malignant hematopoiesis or in mammalian development. We report here that *Tle4* knockout mice are runted and die at around four weeks with defects in bone development and BM aplasia. By two weeks of age, *Tle4* knockout mice exhibit leukocytopenia, B cell lymphopenia, and significant reductions in hematopoietic stem and progenitor cells. *Tle4* deficient hematopoietic stem cells are intrinsically defective in B lymphopoiesis and exhaust upon stress, such as serial transplantation. In the absence of *Tle4* there is a profound decrease in bone mineralization. In addition, *Tle4* knockout stromal cells are defective at maintaining wild-type hematopoietic stem cell function *in vitro*. In summary, we illustrate a novel and essential role for *Tle4* in the extrinsic and intrinsic regulation of hematopoiesis and in bone development.

## Introduction

The bone marrow (BM) is a heterotypic organ that dynamically integrates a variety of signals to modulate both quantitative and qualitative output of hematopoiesis to meet specific needs such as oxygen transport, immunity, and clotting. It is increasingly recognized that the differentiation of blood cells is affected not only by factors intrinsic to hematopoietic stem and progenitor cells (HSPC) but also a variety of cell types in the HSPC niche, including endothelial cells, osteolineage cells, sympathetic neurons, Cxcl12-activated reticular (CAR) cells, and nestin expressing stromal cells [1]. Together, these external and internal regulators work in concert to dynamically respond to physiological demand.

Genetic aberrations in these HSPC can lead to clonal expansions of progenitor cells as in leukemia. One such recurrent genetic mutation occurring in approximately 2% of acute myeloid leukemia (AML) patients is del(9q), which is enriched in patients with the t(8∶21) fusion protein *AML1-ETO* [2]. Previously, we showed that loss of two genes, *Tle1* and *Tle4*, within the commonly deleted region of 9q can cooperate specifically with the *AML1-ETO* fusion product in a zebrafish model of myeloblastic expansion. Moreover, modulation of these genes in cell lines harboring t(8; 21) can influence the proliferative and apoptotic rate of these cells [3]. Additionally, *Tle1* has been shown to be silenced by methylation in a broader set of AML samples, as well as in non-Hodgkin’s lymphoma and diffuse large B-cell lymphomas [4].

The TLE family of genes is a group of highly conserved transcriptional corepressors. The TLE homologue in Drosophila, *Groucho* (Gro), plays a crucial role in multiple developmental processes including neurogenesis, segmentation, and sex determination [5]. Gro also has instructive roles in many signaling pathways including receptor tyrosine kinase/Ras/MAPK, Notch, Wingless (Wg)/Wnt, and Decapentaplegic (Dpp) [6]. Proteins in the *TLE* family can influence transcription by either direct binding to a variety of transcription factors essential to both hematopoiesis and leukemogenesis, including members of the Hes, Runx, LEF1/Tcf, Pax, and Myc families, as well as recruitment of histone deacetylases and methylases, leading to chromatin silencing via condensation over large domains [7]. A combination of these effects likely underlies the ability of this protein family to influence cell fate and malignant transformation. Depending on context, these proteins may behave as either tumor suppressor genes or as facilitators of oncogenesis as in invasive breast cancer and synovial cell sarcoma, respectively [8], [9].

To better understand the role of *Tle4* in development and oncogenesis, we developed a *Tle4* knockout (KO) mouse. *Tle4* KO mice have significant postnatal growth abnormalities, including skeletal and hematological defects. By three weeks of age, KO mice are leukopenic and display specific deficiencies of B cells and HSPC. We show that these defects arise from a combination of both intrinsic and extrinsic defects.

## Materials and Methods

### Generation of *Tle4* null Mice

**Figure 1:**
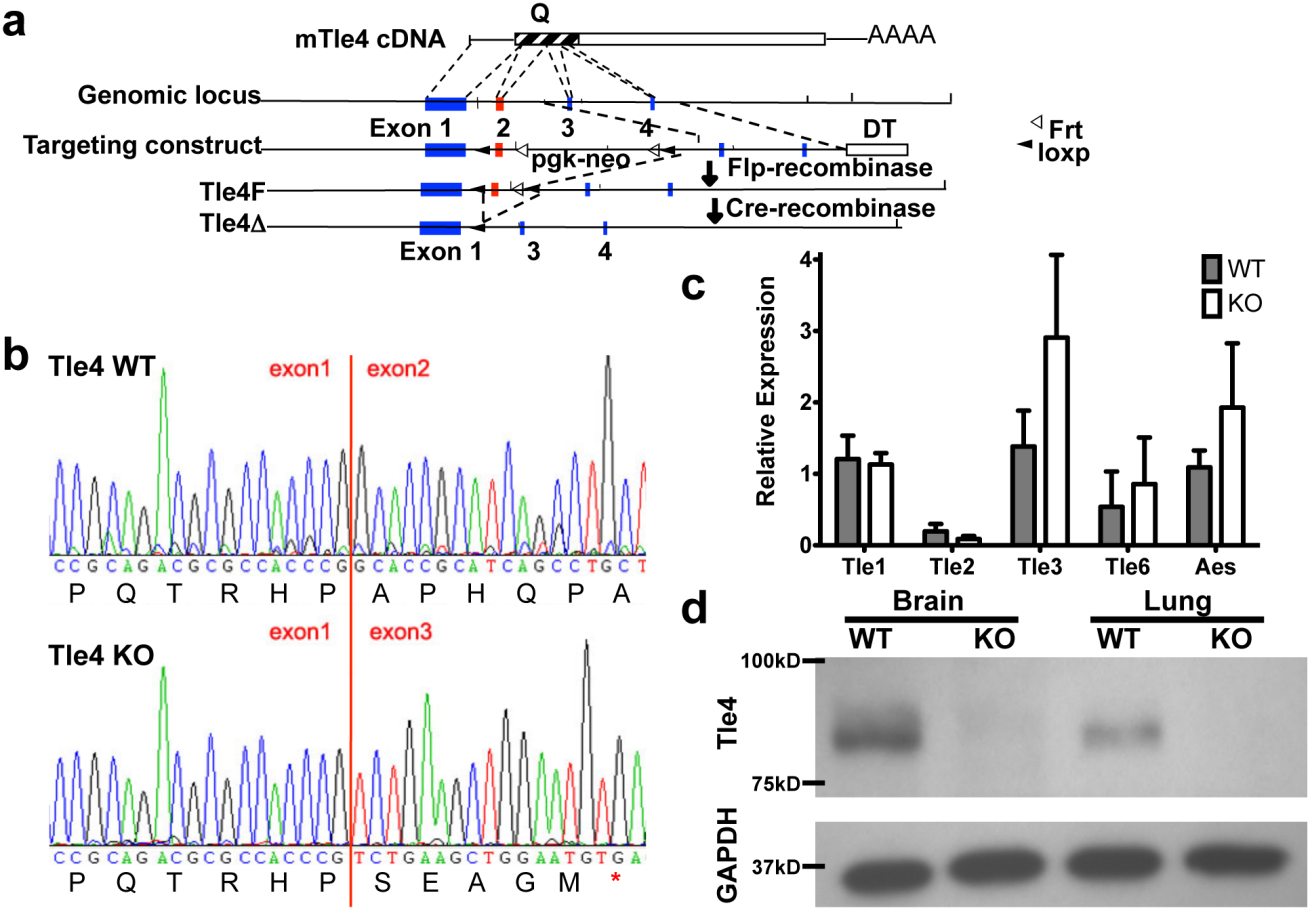
The development of a novel *Tle4* null mouse model. (a) Targeting schema for generation of *Tle4* null animals. Conditional *Tle4* null mice were created by homologous recombination with an original construct containing a pgk-neo positive selection cassette and a Diptheria toxin (Dt) negative selection sequence, The pgk-neo selection cassette was excised between flanking Frt sites (white arrow head) by breeding mice to beta actin-Flp mice, leaving loxp sites flanking exon 2. These conditionally *Tle4* null mice were bred to beta-actin cre mice to generate *Tle4* null mice with a deleted exon 2 between flanking Loxp sites (black arrowheads). (b) RT-PCR showed exon 2 was cleanly excised. Loss of exon 2 creates a frameshift in the cDNA and a truncated non-functional Tle4 peptide. (c) Loss of Tle4 does not significantly affect the expression of other Tle family members as shown by RT-PCR of cDNA from the bone marrow of 2 week old mice. (d) Loss of Tle4 expression in *Tle4* null mice was demonstrated by Western blot with protein from brain and lung.

A conditional *Tle4* null mouse was constructed by targeting LoxP sites to flank exon 2 via homologous recombination using the 129S6/SvEvTac ES cell line (Figure 1a). Resultant mice were crossed with *β-actin: Cre* mice (gift of Susan Dymecki) to delete exon 2 in all tissues. Heterozygote mice were backcrossed to C57BL/6 background for over 6 generations and interbred to generate *Tle4* null mice.

### Whole Mount Staining Of Skeletons to Visualize Cartilage and Calcified Bone

Embryos at embryonic age day 19.5 (E19.5) and one day old newborn pups were euthanized and skeletons subsequently cleared of skin and viscera. Specimens were fixed in 95% ETOH for at least five days, followed by at least two days in 100% acetone to remove adipose tissue. Specimens were then stained in 0.3% Alcian blue, 0.1% Alizarin red in 70% ETOH, and 5% acetic acid for three days. After rinsing in water, specimens were cleared in 2% KOH for 24 hours, 1% KOH/20% glycerol for 5–7 days, 1% KOH/50% glycerol 5–7 days, 1% KOH/80% glycerol 5–7 days, and finally stored in 100% glycerol.

### Terminal Deoxynucleotidyl transferase dUTP nick end labeling Stain (TUNEL)

Paraffin-embedded humeri harvested from two week old *Tle4* WT and KO mice were sectioned for TUNEL staining using the Apoptag kit per manufacturer’s protocol (EMD Millipore, Billerica, MA). Briefly, sections were bathed in Tris buffer with Tween X, followed by proteinase K, peroxidase block, and TdT enzyme treatments. All antibodies used for TUNEL staining were included in the kit and sections were counterstained using Methyl Green.

### BM Transplantation and Homing Assays

BM transplantation was performed by injecting bulk two week old BM or E13.5 fetal liver cells via tail vein into lethally irradiated six to eight week old CD45.1 expressing C57BL/6 recipient mice. Serial transplantation was conducted similarly with pooled BM of primary or secondary recipients for secondary or tertiary transplantation, respectively. For secondary fetal liver transplantation, 1×10^5^ whole BM cells from companion WT CD45.1^+^ mice were included to aid in the post engraftment phase during secondary transplantation. Mice were followed for at least 16 weeks prior to BM and blood analysis by flow cytometry and complete blood count analysis (VetScan HM5, Abaxis, Union City, CA).

To assess homing potential, total BM from three week old KO and WT littermates were pooled by genotype and stained with 1∶500 dilutions of DiD or DiI (Molecular Probes, Inc., Eugene, OR) and 1×10^7^ cells were injected into lethally irradiated WT CD45.1 expressing C57BL/6 mice. After 18 hours post-transplantation, total BM was isolated from recipient mice and analyzed using a FACS Calibur flow cytometer (Becton Dickinson, San Jose, CA). The homing index was calculated as a quotient of the Input Ratio (%KO/%WT) over the Recovered Ratio (%KO/%WT), as described previously [10].

### Flow Cytometry and Cell Sorting

Cells were stained and analyzed on either a FACSCalibur II or LSR II (Becton Dickinson, San Jose, CA). The following anti-mouse antibodies were used for flow cytometry analysis: B220-PE, CD3e-FITC, CD11b-APC, Gr-1-APC-Cy7, CD48-Pacific Blue, CD150-PE-Cy7, CD34, c-Kit-APC, Sca-1-PE, FcγR-PE, CD45.1-Brilliant Violet 570, and CD45.2-Alexa Fluor 700 (all from eBiosciences, San Diego, CA). Cell sorting was performed using a FACS Aria (Becton Dickinson, San Jose, CA). Antibodies for Annexin V and Ki-67 assays were used as per manufacturer’s protocol (Becton Dickinson, San Jose, CA). Gating strategy for HSPC fractions are as follows: (1) LSK: Lineage^Neg^Ckit+Sca1+; (2) CMP: Lineage^Neg^Ckit+Sca1^Neg^ CD34+FcγRIII^low^; (3) GMP: Lineage^Neg^Ckit+Sca1^Neg^ CD34+FcγRIII^high^; (4) MEP: Lineage^Neg^Ckit+Sca1^Neg^ CD34^Neg^FcγRIII^Neg^; (5) CLP: Lineage^Neg^ IL7Rα^high^Ckit+Sca1^low^; ProB Frac A: B220^low^CD43^High^BP1^Neg^HSA^Neg^; ProB Frac B: B220^low^CD43^High^BP1^Neg^HSA^Pos^; ProB Frac C: B220^low^CD43^High^BP1^High^HSA^Pos^; ProB Frac D: B220^High^CD43^Neg^IgM^Neg^; ProB Frac E: B220^High^CD43^Neg^IgM^Mid^; ProB Frac F: B220^High++^CD43^Neg^IgM^High/Mid^.

### Stromal co-culture and colony forming unit assays (CFU)

For the methylcellulose colonies, whole BM from 2 week old WT and KO mice were lineage depleted using Biotin lineage antibody cocktail (B220, CD3, CD4, CD8, Mac-1, Gr-1 and Ter119) and Streptavidin magnetic microbeads (Miltenyi Biotec, Cambridge, MA). Cells were subsequently sorted by flow cytometry using the following fluorescent antibodies: PE-Cy7 lineage cocktail, c-Kit-APC, Sca-1-PE (eBiosciences, San Diego, CA). 1×10^3^ sorted LKS cells from each cohort were plated in M3434 Methocult (Stem Cell Technologies, Vancouver, CA) according to the manufacturer’s protocol. Colonies were manually counted 7 days and 14 days after plating for serial replating and colony differentiation assays, respectively.

Stromal co-culture was performed as previously described [11]. Briefly, cells from crushed bones of three day old KO or WT littermates were grown for two weeks in 20% FBS in α-MEM supplemented with Penicillin/Streptomycin. After reaching confluence, media was changed to osteogenic media containing 100 µM β-glycerophosphate, 2.84 µM ascorbic acid, and 10 nM dexamethasone. Three weeks after osteogenic induction, 100 Lin^−^ c-Kit^+^ Sca-1^+^ CD34^−^ CD48^−^ CD150^+^ cells were sorted directly onto osteoblastic stromal cells, and allowed to expand for 4 weeks. Flow analysis for c-Kit and Sca-1 expression was performed two weeks after initiation of co-culture. After four weeks of co-culture, 1×10^3^ cells from each co-culture assay sample were seeded in Methocult 3434 (Stem Cell Technologies, Vancouver, Canada) and assessed for colony forming units by manual count one week later.

### Western Blot, qRT-PCR

Protein lysates of bone and osteoblast-like cells from WT and KO samples of above co-culture experiments, as well as from brain and lung from WT and KO animals were prepared for western blot as previously described [12]. Western blots were probed with an antibody to TLE4 (ab64833, Abcam, Cambridge, MA), GAPDH (Abcam), murine stem cell factor (Scf) (Santa Cruz Biotechnology Inc., Santa Cruz, CA) and β-actin (Santa Cruz Biotechnology Inc.), followed by horseradish peroxidase conjugated secondary antibodies (Santa Cruz Biotechnology, Inc.) and visualization by chemoluminescence (Pierce ECL, Thermo Scientific, Rockford, Ill).

qRT-PCR for Tle1, Tle2, Tle3, Tle6, and Aes were performed using TaqMan Assays (Life Technologies, Grand Island, NY).

### Statistics

The Student’s unpaired *t*-test was used for most analyses. A paired Student’s *t*-test was used for the homing index. Significance was set at *P*<0.05.

### Ethics Statement

This study was carried out in strict accordance with the recommendations in the Guide for the Care and Use of Laboratory Animals of the National Institutes of Health. The protocol was approved the Massachusetts General Hospital Institutional Animal Care and Use Committee (IACUC, permit #2004N000277). Animals were sacrificed according to institutional guidelines by use of CO_2_ asphyxiation. All efforts were made to minimize suffering.

## Results

### Verification of *Tle4* knockout

Exon 2 of *Tle4* was deleted by crossing *β-actin: Cre* mice with conditional *Tle4* null mice containing *LoxP* sites flanking the exon (Figure 1a). Excision of exon 2 removes a lysine zipper motif critical to the function of the Q-oligomerization domain [13] and results in a frameshift leading to a truncated protein of 13 amino acids. Correct targeting and exon 2 deletion was verified by Southern blot (not shown). Successful recombination and deletion of exon 2 was verified by cDNA sequencing (Figure 1b). Levels of other Tle protein family members did not significantly differ between WT and KO animals, as verified by qPCR (Figure 1c). Western blot of both brain and lung (Figure 1d) confirmed that genetic ablation of *Tle4* resulted in the absence of *Tle4* protein expression.

### *Tle4* null mice have growth defects and skeletal abnormalities

**Figure 2:**
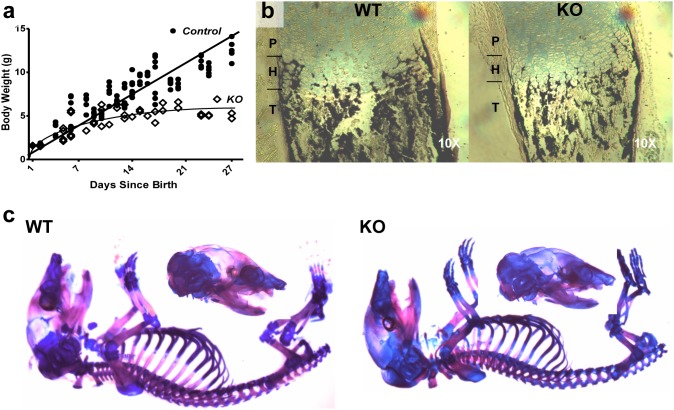
*Tle4* null mice exhibit growth retardation and under-mineralization of bone. (a) *Tle4* null mice are born of similar size to wild-type littermates, but exhibit severe growth retardation after birth. (b) One day old *Tle4* null mice (KO) have decreased mineralization of the trabeculae and cortical bone in the tibiae compared to wild-type littermates (WT) as shown by Von Kossa staining. (c) Alizarin Red/Alcian Blue staining for ossified bone (red) and cartilage (blue) of 1 day old wild-type (WT) and *Tle4* null (KO) skeletons shows decreased mineralization in *Tle4* null mice of both membranous bone (skull) and endochondral bone (vertebrae and long bones).

*Tle4* null mice appear grossly normal at birth without obvious organ abnormalities. However, their growth plateaus by the second week of life compared to littermates that are wild-type (WT) or heterozygous (HET) for *Tle4* (Figure 2a). *Tle4* null mice become moribund and perish by four weeks of life. No phenotypic differences were observed between WT and HET mice. WT littermate mice served as controls for all following experiments.

**Figure 3:**
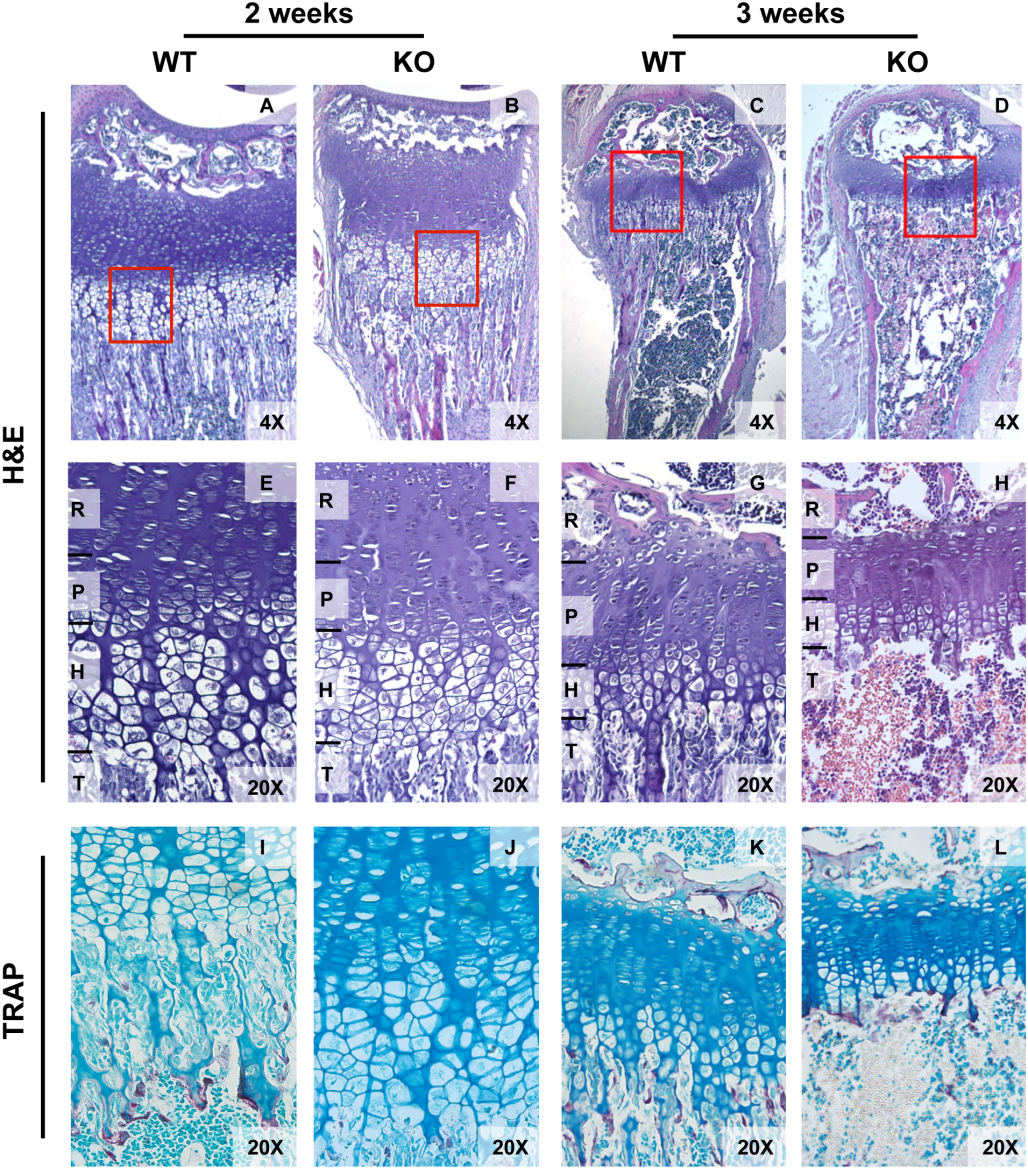
Progressive bone marrow hypoplasia with defective ossification, loss of trabecular bone and thinning of cortical bone is seen by 3 and 4 weeks in *Tle4* null mice. A–D. Hematoxylin and Eosin (H&E) staining of tibiae of 3 and 4 week old WT and *Tle4* null (KO) mice demonstrate multiple abnormalities in *Tle4* null mice including progressive pancytopenia of the bone marrow (BM), loss of trabecular bone (T), and thinning of the cortical bone layer (C). A higher power view shows a thinner proximal tibial growth plate in *Tle4* null mice with a decrease in thickness of the resting (R), proliferative (P), and hypertrophic (H) zones and near complete loss of the trabeculae. E–H. Tartrate-resistant acid phosphatase (TRAP) staining (pink) demonstrates osteoclasts clustering under the hypertrophic zone at the boundary of the bone marrow cavity in *Tle4* null mice at 3 and 4 weeks of age.

Decreased calcification, as well as thinning of the cortical bone and decreased trabecular bone, was evident by Von Kossa staining of tibiae in one day old KO as compared to WT mice (Figure 2b). Alizarin Red and Alcian Blue staining of calcified bone and cartilage confirmed a significant reduction in the degree of calcification throughout the skeleton of one day old KO mice compared to WT littermates despite no significant differences in overall body size or weight (Figure 2a). By 21–28 days of age, trabecular bone was almost absent in KO tibiae and femurs and abnormal growth plates were present. Though similar at 2 weeks, the growth plates of *Tle4* null mice were much thinner with shorter columns of cells in both the proliferating and hypertrophic zones by 3 weeks of age (Figure 3a–h). At three weeks of age, tartrate-resistant acid phosphatase (TRAP) staining [17] demonstrated osteoclasts underneath the growth plate in KO mice that might be contributing to trabecular resorption (Figure 3i–l) [14]. These data indicate that *Tle4* null animals have developmental defects in the skeletal system, fail to thrive, and subsequently die prematurely by 4 weeks of life.

### *Tle4* null mice have hematological abnormalities by two weeks of age

**Figure 4:**
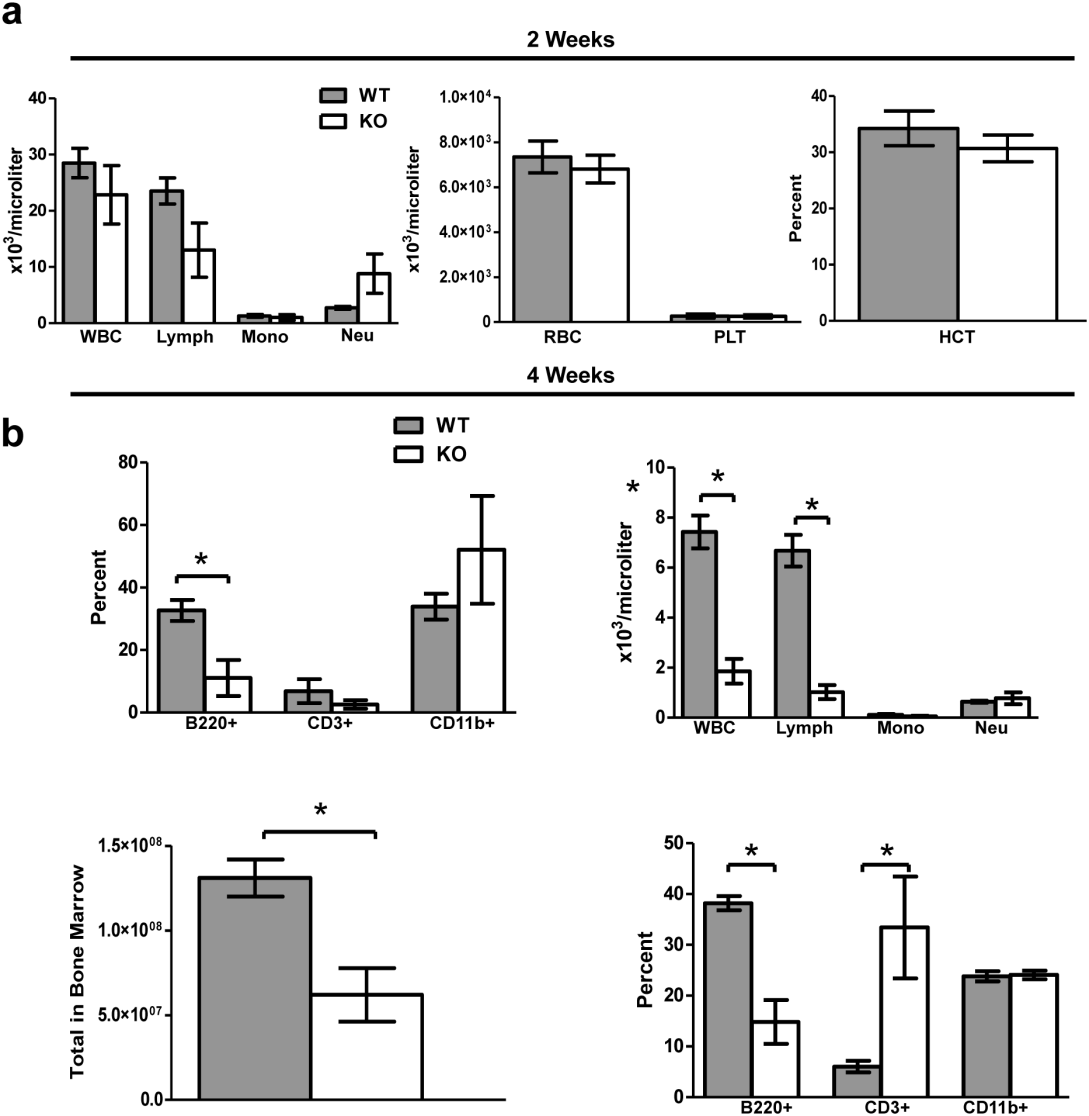
Peripheral blood counts are normal in *Tle4* null mice at 2 weeks of age, but significant abnormalities are seen in peripheral blood and bone marrow at 4 weeks of age. (a) At 2 weeks of age there was no significant difference in any of the cell compartments in the peripheral blood of *Tle4* null mice (KO) as compared with normal control littermates (WT). However, by 4 weeks of age *Tle4* null mice exhibit a marked leukopenia (decreased WBC) and lymphopenia in the peripheral blood, that primarily affects the B-cells (B220+ cells), and not T-cell (CD3+) or myeloid cells (CD11b+). (b) *Tle4* null mice exhibit severe bone marrow aplasia with a 2-fold decrease in bone marrow cellularity. Within the remaining population of lymphoid cells, B-cell development appears particularly affected with a significant decrease in the percentage of B-cells (B220+) and relative increase in the percentage of T-cells (CD3+).

At two weeks of age, there were no significant differences in absolute numbers of all blood cell lineages between KO and WT mice as determined by complete blood count (CBC) (Figure 4a). However, immunophenotypic analysis of peripheral blood illustrated a significant reduction in the frequency of B220^+^ cells, but no significant difference in CD3^+^ or CD11b^+^ cells (Figure 4a). BM of two-week-old KO mice exhibited a two-fold reduction in total cellularity (Figure 4b), significant reductions in the frequency of B220^+^ cells, and an increase in the frequency of CD3^+^ cells (Figures 4b). By four weeks of age, KO animals had progressed to severe leukopenia, and more specifically lymphopenia, in the blood (Figure 4b).

**Figure 5:**
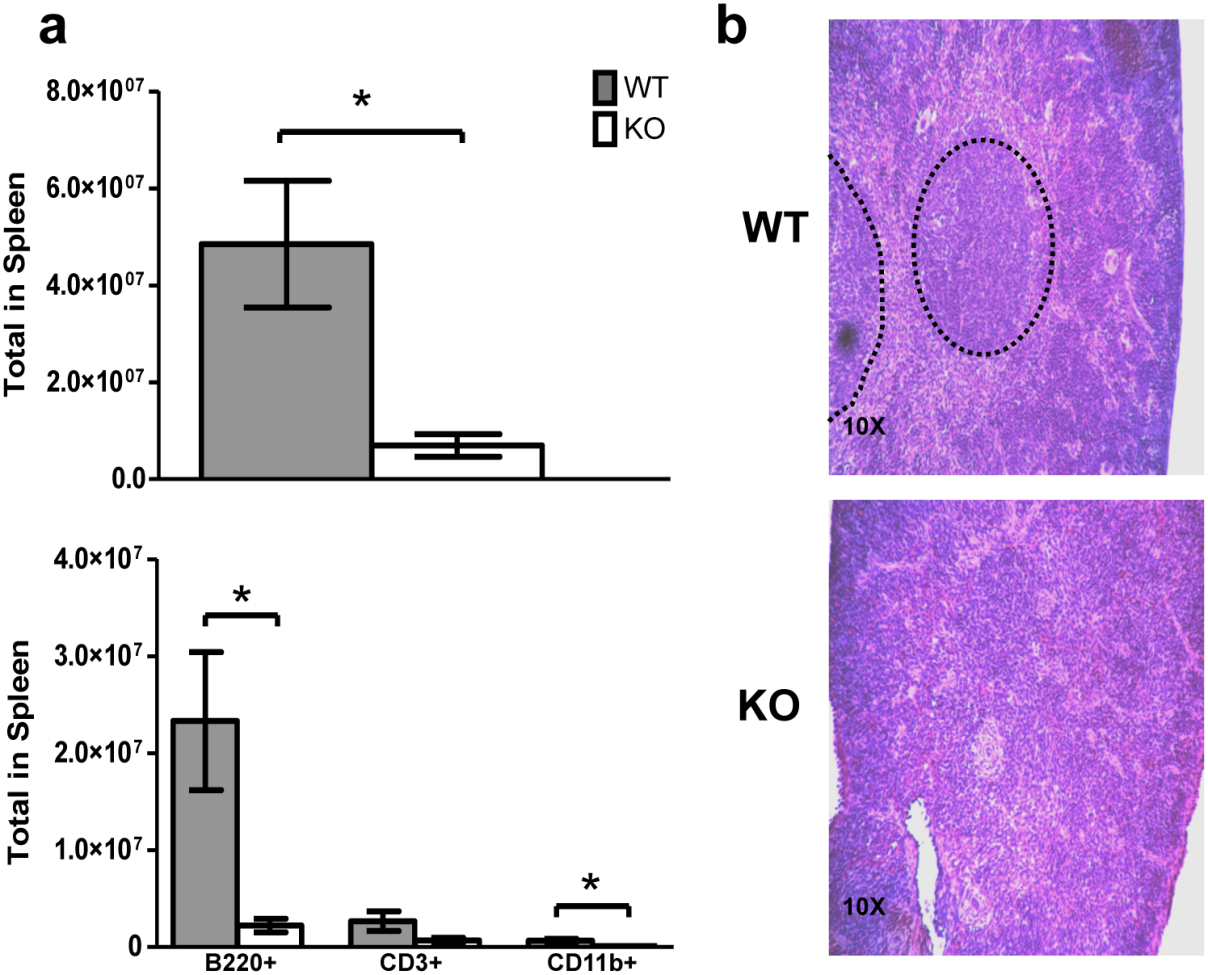
*Tle4* null mice develop splenic atrophy with abnormal splenic architecture. (a) At three weeks of age there is marked splenic atrophy and decreased cellularity especially of the major B-cell (B220+) compartment. (b) H&E staining of the spleen reveals an absence of splenic follicles (dashed oval) in two week old *Tle4* null mice (KO). (n = 3–4 per genotype; mean +/−SEM; *: *P*<.05).

**Figure 6:**
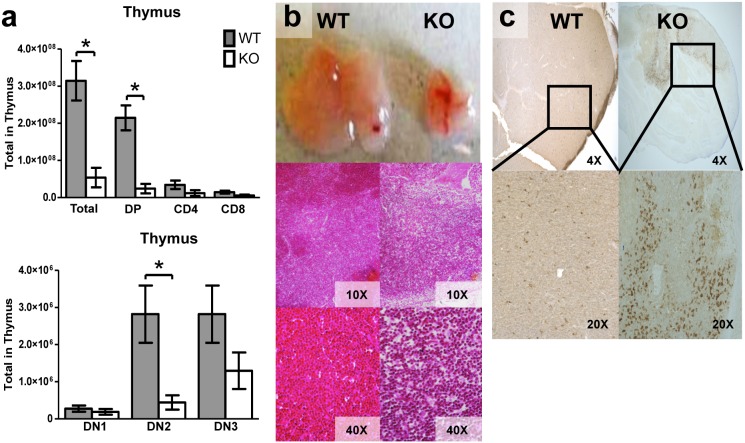
*Tle4* null mice develop thymic atrophy with a block in T-cell differentiation. (a) There is a dramatic decrease in total thymocytes in 3 week old Tle4 null mice as compared to wild-type littermates. The majority of this decrease is due to loss of double positive CD4^+^CD8^+^ cells. Within the double negative (DN) T progenitor populations there appears to be a block between DN1 (CD44+CD25−) and DN2 (CD44+CD25+) with a significant decrement in DN2 cells and an insignificant decrease in DN3 (CD44−, CD25+) cells. (n = 3–4 per genotype; mean +/−SEM; *: *P*<.05). (b) The thymus of 3 week old Tle4 null mice is atrophied with a loss in the demarcation between cortex and medulla as seen by H&E. (c) TUNEL staining demonstrates thymic apoptosis in Tle4 mice.

By three weeks of age, KO mice also developed abnormalities in the spleen and thymus. Splenic cellularity was significantly reduced in KO mice (Figure 5a), most likely due to significant reductions of total B220^+^ lymphocytes (Figures 5a). Furthermore, the splenic architecture was disrupted in KO mice with an almost complete absence of splenic follicular zones (Figure 5b). The total cellularity of the thymus and T cell subsets in three week old mice were significantly reduced (Figure 6a). The KO animals also had significant thymic atrophy compared to WT mice without a clear demarcation between the cortex and medulla (Figures 6b). TUNEL staining of the thymus harvested from three week old KO mice demonstrated increased thymic apoptosis (Figure 6c). In summary, deletion of *Tle4* resulted in B-cell lymphopenia in peripheral blood and decreased cellularity in hematopoietic organs.

### Loss of *Tle4* has deleterious effects on HSPC

**Figure 7:**
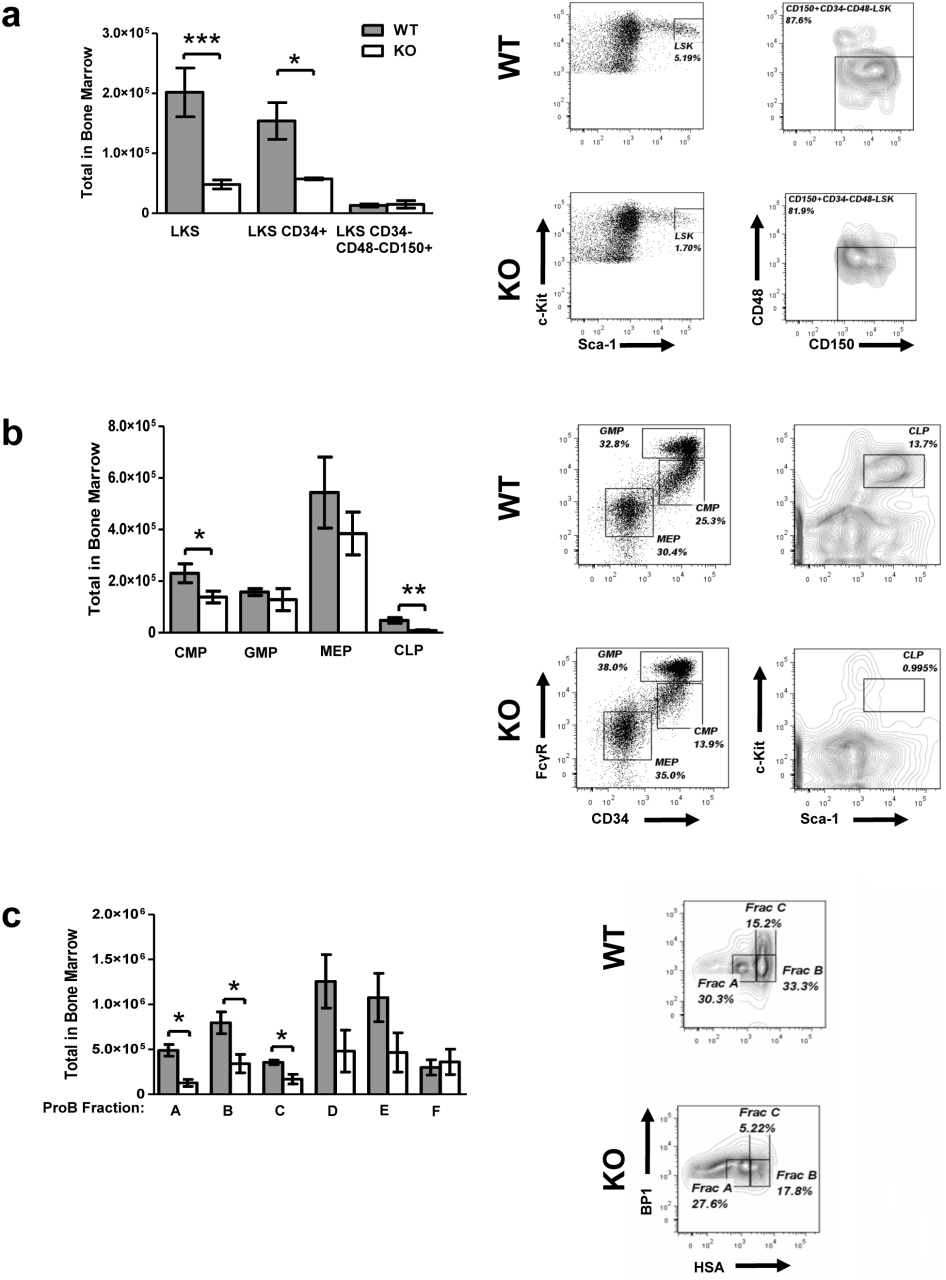
*Tle4* null mice have significant aberrations in hematopoietic stem and progenitor cells (HSPC). Bar graphs with representative flow cytometry plots in two week old littermates (a) show significant loss of LSK and LKS CD34+ cells, though the most immature long-term HSC population (LKS CD34^+^CD48^–^CD150^+^ HSC) is relatively preserved. (b) Examination of CMP, GMP, MEP, and CLP progenitor fractions demonstrated significant decreases in CMP and CLP populations. (c) Amongst the Pre/Pro B cell progenitors the decrement is most prominent in the early Fractions A through C. (n = 3–8 per genotype; mean +/− SEM; *: *P*<.05, **: *P*<.001, ***: *P*<.0001). See methods for gating strategy of stem and progenitor cell compartments.

*Tle4* null mice were found to have an almost four-fold reduction in the total number of Lin^−^ c-Kit^+^ Sca-1^+^ (LKS) cells and a three-fold reduction in CD34^+^ LKS cells (Figure 7a). Interestingly, the total number of long-term HSC, defined by surface expression of SLAM markers CD150^+^ and CD48^−^, were not reduced in KO mice despite the five-fold reduction in total BM leukocytes at this age (Figure 7a). However, total numbers of common myeloid (CMP) and more so common lymphoid progenitors (CLP) were significantly reduced in *Tle4* null mice (Figure 7b). Finally, *Tle4* null mice also have significant reductions in Pre/ProB fractions A through C (Figure 7c). *Pax5*, which derives its repressive activity via interaction with *Tle4*, is necessary for progression through the Pre/ProB checkpoints. Deletion of *Pax5* results specifically in a block in B cell development between Fractions B (early pro-B) and C (late pro-B) [15], [16]. Therefore, while some component of the *Tle4* null ProB phenotype may be derived from deregulated Pax5 signaling, it also appears that loss of *Tle4* led to more deleterious effects on B cell development than mere phenocopy of *Pax5* deficiency. Taken together, loss of *Tle4* appears to decrease the frequency of LKS cells and especially B lymphoid progenitors, but not of LKS SLAM cells.

**Figure 8:**
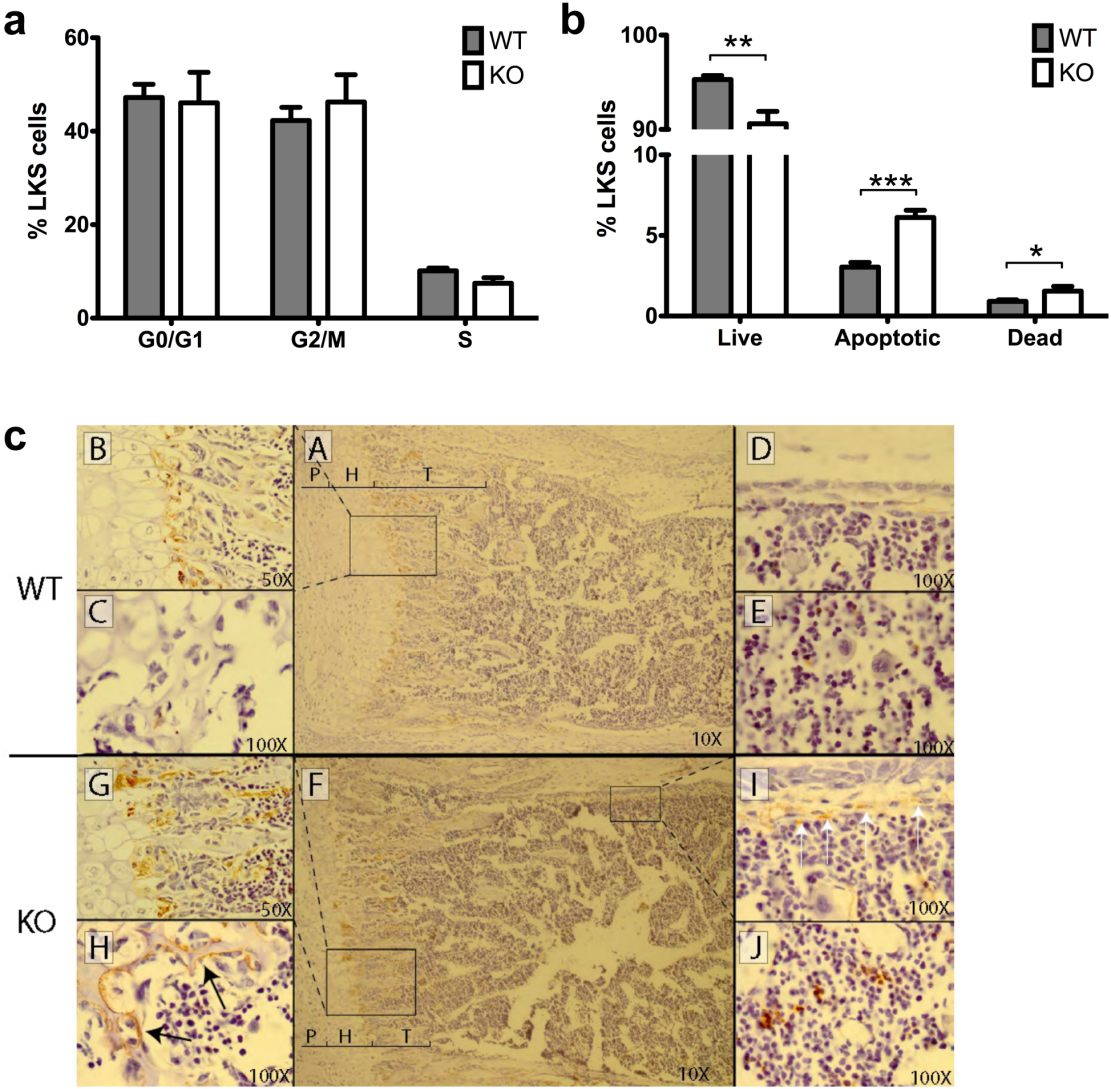
The decrease in LKS cells in Tle4 null mice is due to an increase in apoptosis and cell death rather than a decrease in proliferation and is accompanied by abnormalities of the bone marrow stroma. (a) LKS cells isolated from the bone marrow of two week old mice show no difference in cell cycle distribution. (b) There is however an increase in apoptotic and dead LKS cells in the Tle4 null mice. (c) TUNEL staining of the growth plate of the femur in two week old mice marks the normal zone of cell death between the hypertrophic (H) layer and forming trabecular (T) bone (A, B) with an increase in staining in Tle4 null mice (F, G). Lacunae in the epiphysis are lined with periosteal cells undergoing apoptosis in Tle4 null mice (H), but was not seen in wild type (WT) littermates. Similar periosteal cells undergoing apoptosis and stained by TUNEL are seen under the cortex of diaphyseal bone in Tle4 null mice (I) but absent in wild-type mice (D). An increase in TUNEL staining is also observed in cells of the bone marrow in Tle4 null mice (J) as compared to wild-type bone marrow (E).

To further typify the above-described difference in LKS frequency between WT and KO mice, additional studies were done to examine cell proliferation and apoptosis within LKS populations. There is no difference in cell proliferation and cell cycling between two-week old WT and KO LKS, as indicated by flow cytometry analysis of Ki-67 (Figure 8a). Interestingly, however, Annexin V analysis showed LKS cells from two-week old KO mice exhibiting significantly reduced viability and increased apoptotic and dead cells (Figure 8b). This suggests the decrease in LKS frequency in KO mice may be due to Tle4-dependent increased apoptosis rather than changes in cell proliferation or cycling.

### Co-culture of wild-type HSC on *Tle4* null stromal cells impairs long term colony forming ability

Given the critical importance of the BM niche on hematopoiesis, we hypothesized that the abnormalities in the skeletal or stromal compartments of the BM were contributing to defective hematopoiesis [17], [18]. This was confirmed by TUNEL staining of paraffin-embedded humeri harvested from two-week old WT and KO mice (Figure 8c). Compared to WT, various compartments of KO bone exhibited increased TUNEL staining, including the periosteal cells lining lacunae in the epiphysis (Figure 8c, panel C vs H) and periosteal cells under the cortex of the diaphyseal portion of the humerus (Figure 8c, panel D vs I). Moreover, TUNEL staining was more extensively pervasive throughout trabeculae and bone marrow in KO compared to WT mice (Figure 8c, panel B vs G, E vs J). This further suggested the possibility that the hematopoietic abnormalities seen in KO mice might be at least in part due to the absence of support from the osteoblastic niche.

To further elucidate whether impaired HSC maintenance by KO BM was dependent on stromal cell irregularities, western blots using whole bone lysates from two week old WT and KO mouse were performed using antibodies against *Scf*, a known factor involved in the maintenance of HSC [1] [27]. Though some KO mice displayed a complete lack of Scf expression, others showed modest protein levels (results not shown). The considerable animal to animal variation suggests that down-regulation of *Scf* expression is not a direct effect of Tle4 loss, but might reflect a loss of an *Scf* producing cell type in *Tle4* null mice. Other factors may account for the impairment of HSC maintenance.

**Figure 9:**
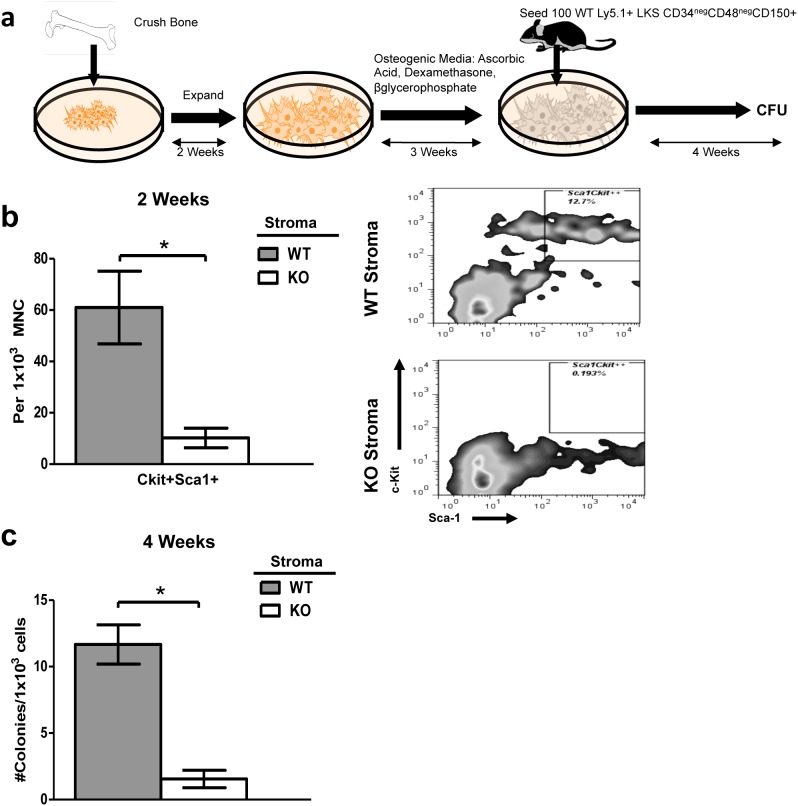
Co-culture of wild-type HSC on *Tle4* null stromal cells impairs long term colony forming ability. (a) Schematic of co-culture assay. Briefly, stromal cells were isolated from bones of 2 week old WT and KO animals and plated in aMEM with dexamethasone, ascorbic acid, and vitamin D_3_ as described in Methods. WT LKS were plated and used in LTC-IC experiments (b) Bar graph showing a dramatic decreased frequency of Sca-1^+^c-Kit^+^ cells after two weeks of co-culture on the stroma from Tle4 null mice with representative flow plots (n = 2 biological replicates per genotype, in triplicate *: *P*<.01). (c) Long term culture-initiating cell assay (LTC-IC) after four weeks of co-culture with WT or KO stroma showed relatively few colonies obtained after culturing on Tle4 null bone marrow stroma (n = 2 biological replicates, each in 9-plicate, seeded in duplicates for CFU; *: *P*<.0001).

Since *Tle4* null animals do not survive long enough to serve as bone marrow recipients of wild-type HSPC, we adapted a co-culturing assay to determine whether HSC-extrinsic factors may have influenced the observed hematological phenotype [11]. Wild-type LKS cells from two week old mice were co-cultured on WT or KO stroma cells derived from 3 day old mice for two weeks (Figure 9a). Non-adherent cells were harvested and analyzed for expression of c-Kit and Sca-1. In WT co-cultures, 6–15% of recovered cells were positive for both markers. In stark contrast, less than 1% of cells recovered from WT LKS cells plated on KO stromal co-cultures were C-kit^+^ Sca-1^+^ (Figure 9b). Long-term co-culture experiments plated in methylcellulose revealed an even more pronounced effect. Cells recovered from WT co-cultures exhibited an average of 10-fold more colonies than their KO counterparts (Figure 9c). Some KO co-cultures failed to exhibit any colony forming ability. Thus, *Tle4* null stromal cells cannot maintain and support HSPC growth as efficiently as WT stromal cells.

### *Tle4* null HSPC are intrinsically impaired in B cell differentiation and exhaust after serial transplantation

**Figure 10:**
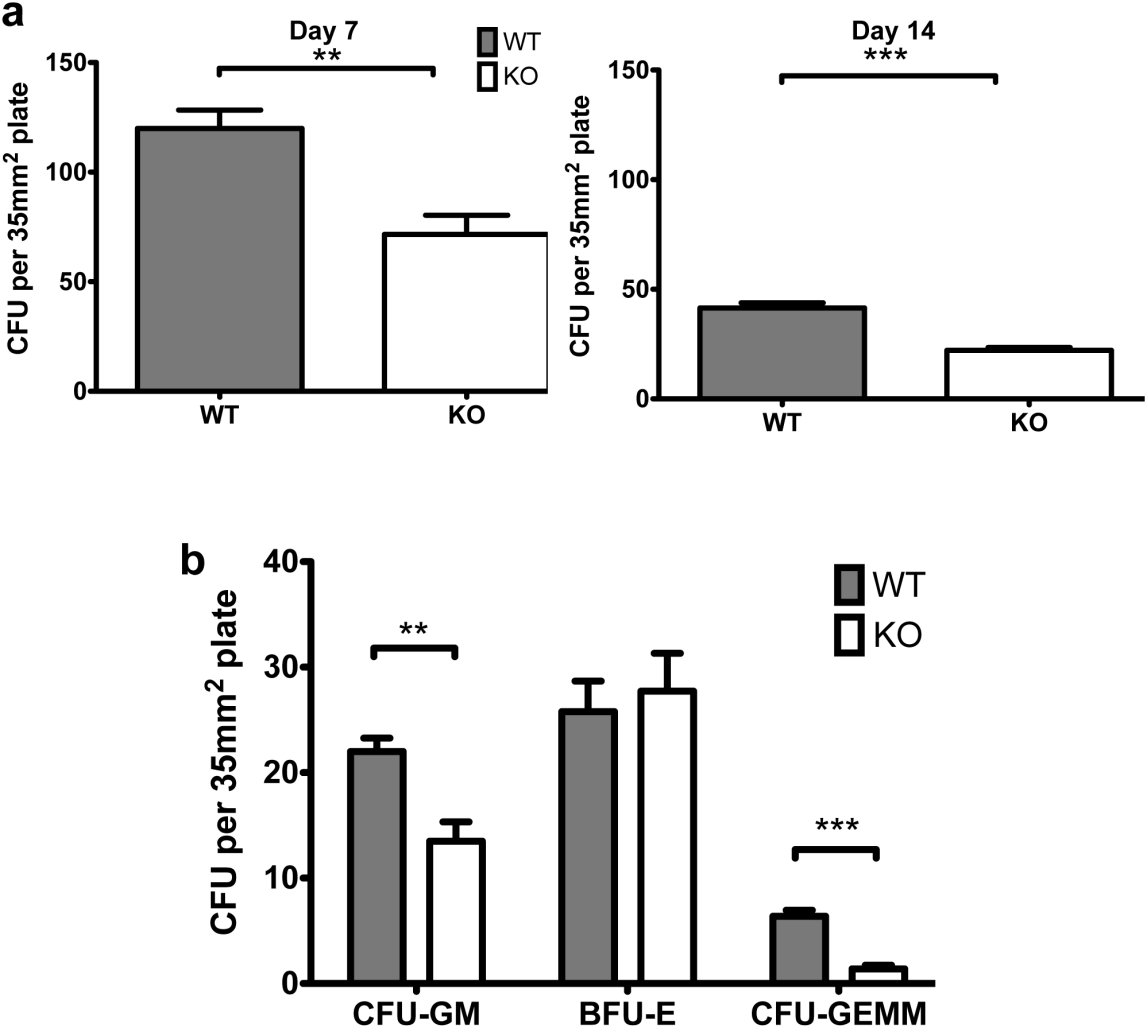
LKS cells from TLE4 null mice are impaired in colony forming unit ability. (a) The number of colonies in methyl cellulose originating from 1000 LKS cells from TLE4 null mice were fewer compared to wild-type LKS cells on Day 7 and again after replating on Day 14. (n = 4 biological replicates per genotype, in triplicate) (b) LKS cells from Tle4 null mice form fewer CFU-GM and CFU-GEMM colonies but similar BFU-E colonies compared to wild-type. (n = 5 biological replicates per genotype, in triplicate) (**: *P*<.01, ***: *P*<.0001).

Due to the progressive leukopenia in primary *Tle4* null mice and the extrinsic effects noted above, we tested the colony-forming ability of sorted LKS cells from 2 week old WT or *TLE4* null mice. After seven days in methylcellulose, *TLE4* null LKS were significantly less efficient at forming colonies than WT LKS cells, suggesting intrinsic defects in HSC and HSPC and impaired HSC-self renewal in *Tle4* null mice (Figure 10a). These observations were further reinforced by a continued decrease in the ability of KO LKS cells to produce colonies when serially replated in methylcellulose at 14 days (Figure 10a). Additionally, these colonies were also typified as granulocyte/macrophage-forming (CFU-GM), erythroid-forming (BFU-E), or granulocyte/erythrocyte/monocyte/megakaryocyte-forming (CFU-GEMM) colony forming units to identify any *Tle4*-dependent HSC-intrinsic effects on progenitor differentiation. After 13 days in methylcellulose culture, sorted LKS cells from KO were less efficient in forming CFU-GM and CFU-GEMM compared to their WT counterparts (Figure 10b).

**Figure 11:**
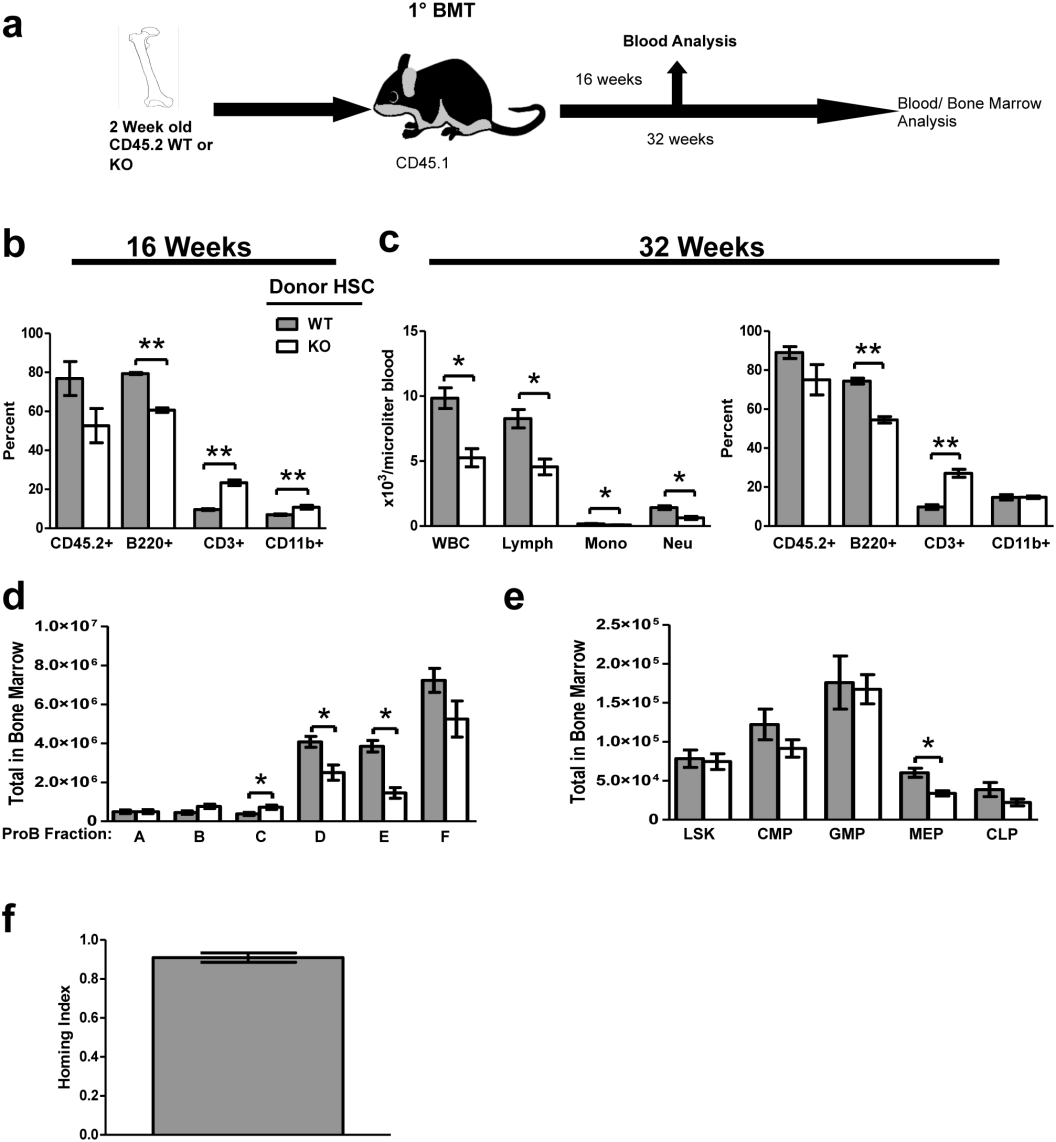
*Tle4* null mice have cell-intrinsic defects in HSPC self-renewal and B-cell development. Bone marrow transplants of wild-type (WT) or Tle4 null (KO) bone marrow from 2 week old mice into normal recipients were performed to evaluate cell intrinsic defects. (a) Schematic of BMT experimental design. (b) Analysis of peripheral blood at 16 after transplant by flow cytometry and CBC demonstrated insignificant differences in engraftment as measured by the percent CD45.2+ cells, but impaired B-cell numbers (B220+) with relatively increased T-cells (CD3+) and myeloid cells (CD11b+). (c) 32 weeks after transplant mice receiving Tle4 null bone marrow developed leukopenia (decreased WBC) primarily accounted for by a decrease in lymphocytes (c, left panel), which by immunophenotyping represented a decrease in B-cells (B220+). (n = 9–10 per transplant group, *: *P*<.005 **: *P<.*0001). (d) Analysis of B-cell differentiation in the BM of recipient mice 32 weeks after transplant by quantitation of ProB Fractions shows an increase in Fraction C, but decreased Fractions D and E indicating a relative block in differentiation between Fractions C and D (n = 5 per genotype, *: *P*<.05). (e) Analysis of bone marrow 32 weeks after transplant showed no significant differences between KO and WT recipients in the absolute number of LKS, CLP, CMP, or GMP compartments, but did show a decrease in MEPs in KO recipient mice. (f) Competitive homing 18 hours after transplantation using whole BM from two week old WT and KO littermates showed no defect in homing ability (n = 3). The homing index is represented as: Input/Output or 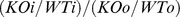.

To further test this effect, we performed *in* *vivo* transplantation assays using BM from 2 week old animal or fetal liver hematopoietic cells to determine whether loss of *Tle4* intrinsically affected HSC self-renewal and repopulation efficiency. Whole BM from two-week-old CD45.2^+^ KO and WT animals was isolated and transplanted into lethally irradiated allogeneic WT mice expressing CD45.1 (Figure 11a). Given our observation of non-significant differences in LKS SLAM populations, representing long-term HSC, between WT and KO BM, equal numbers of cells from donor KO and WT BM were transplanted into recipient mice. Sixteen weeks after BM transplant (BMT), recipients of KO and WT BM showed non-significant differences in donor chimerism in blood (Figure 11b). However, FACS analysis of peripheral blood showed that recipient mice of KO BM had significant reductions in the frequency of B cells, with concomitant increases in both T and myeloid cell frequencies (Figure 11b). At 32 weeks after transplant, CBC analysis of KO BM recipients demonstrated the development of significant leukopenia, especially a decrease in myeloid and lymphoid lineages (Figure 11c, left panel). Immunophenotypic analysis showed that the leukopenia was mostly due to a decreased frequency of B cells (Figure 11c, right panel). Fractionation of B cell precursors in recipients 32 weeks post-transplant showed a reduction in the absolute number of Fraction D and E cells and an increase in Fraction C before progression to fraction D (Figure 11d). This suggests that transplanted *Tle4* null BM cells have a block in B cell development at the late pro-B to pre-B-cell stage, more restricted than seen in situ with Tle4 null mice, but still distinct from the early pro-B to late pro-B block reported with *Pax5* deficiency [15], [16], [19].

Analysis of HSPC in recipients 32 weeks after transplant, which has been shown to reflect long term repopulating activity of HSC [32], revealed no significant differences between KO and WT recipients in the absolute number of LKS, CLP, CMP, or GMP compartments. However, MEPs were significantly reduced in KO recipient mice (Figure 11e). A competitive homing experiment revealed that at 18 hours after transplant, KO HSC were able to home to the recipient niche as efficiently as WT cells (Figure 11f), indicating the observed abnormalities were not due to homing defects of these cells. These findings suggest that BMT with *Tle4* null HSC results in leukopenia, specifically B lymphopenia arising from partial blocks in ProB development. However, KO donor BM retained normal frequencies of most HSPC fractions after transplantation into a normal BM niche.

**Figure 12:**
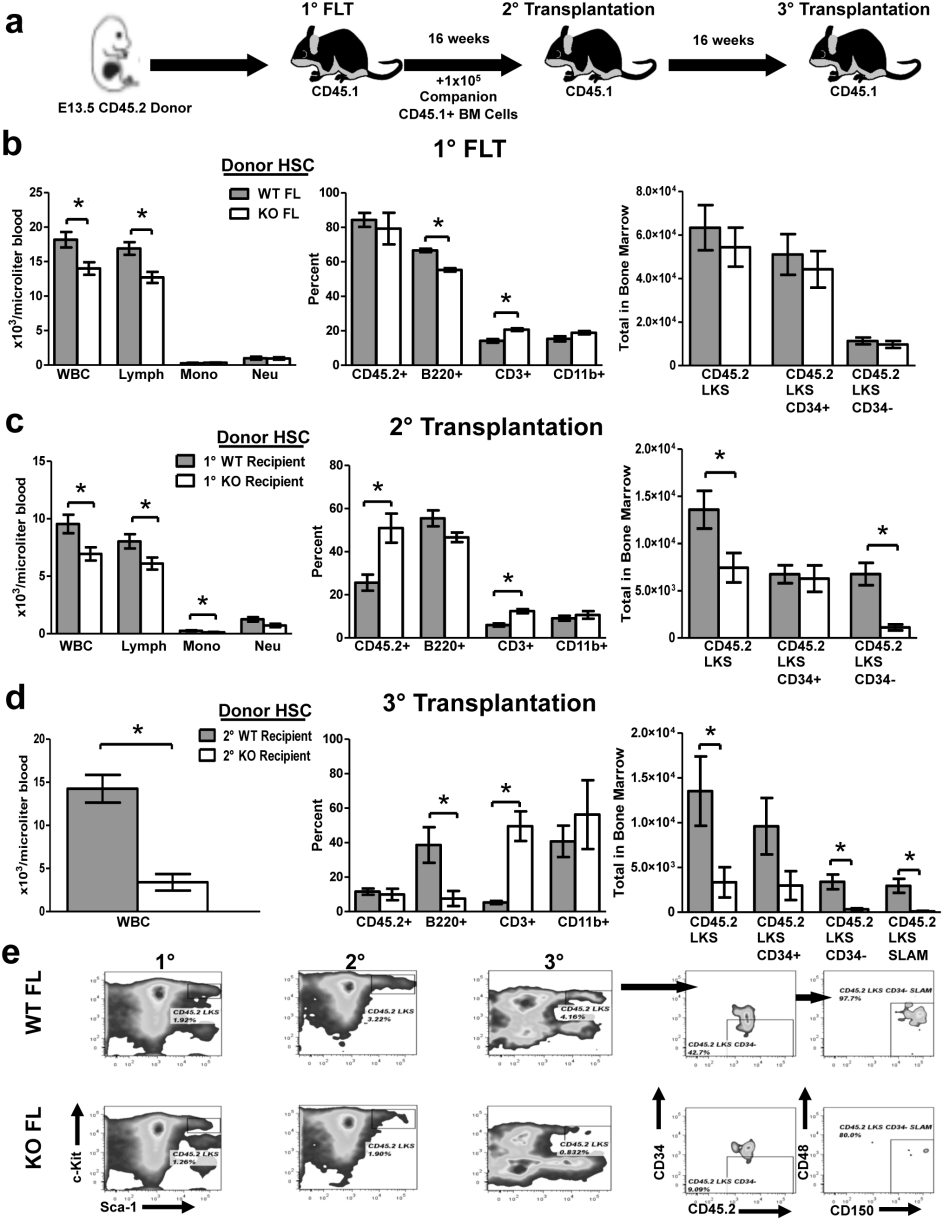
*Tle4* null fetal liver HSCs have impaired B cell development and exhaust with serial transplantation. (a) Schematic of serial FL transplantation experimental design. (b) Peripheral blood and LKS analysis of FL transplant recipients 16 weeks after transplant revealed peripheral leukopenia (WBC) and specifically B cell (B220+) lymphopenia in the absence of Tle4 with no difference in LKS, LKS, CD34+, LKS CD34− populations (n = 10 per genotype for blood analysis, n = 6–7 per genotype for LKS analysis, ***: *P*<.0001). (c) Peripheral blood and LKS analysis 16 weeks after secondary transplantation also indicates leukopenia and lymphopenia in Tle4 null cells, but at this time also a significant decrease in LKS and LKS, CD34− populations (n = 5 KO recipients, n = 10 WT recipients for blood analysis, n = 5 mice per group for LKS analysis, *: *P*<.05 **: *P*<.001). (d) Peripheral blood analysis and LKS analysis 16 weeks after tertiary transplantation again showed leukopenia, B-cell lymphopenia, and a profound decrease in all HSC containing populations (n = 4 per genotype for blood and LKS analysis, *: *P*<.05, **: *P*<.01, ***: *P*<.001). (e) Representative flow cytometry plots showing progressive loss of *Tle4* null HSCs over successive transplantation.

To test self-renewal capacity of KO versus WT HSC while excluding confounding effects possibly due to prior exposure to a defective *Tle4* null BM niche, we performed serial transplantations of fetal liver hematopoietic cells (FL) of E13.5 WT and KO CD45.2^+^ fetuses (Figure 12a). Recipients of KO and WT FL primary transplants exhibited peripheral leukopenia and specific B cell lymphopenia (Figure 7b, left and middle panels), recapitulating the phenotype observed in recipients of KO BM in prior BMT experiments (Figure 11c and 11d). Additionally, there were no differences in the frequency of HSC between primary recipients of KO or WT FL (Figure 12b, right). Upon serial transplantation of FL into secondary transplant recipients, we again saw highly significant leukopenia and lymphopenia 16 weeks after transplant in KO recipient mice (Figure 12c, left). Interestingly, secondary KO FL recipients also exhibited a significant reduction in *Tle4* null-derived LKS (Figure 12c, right). Moreover, these LKS cells present at secondary transplant had high expression of CD34, with few CD34^−^ LKS cells, while WT recipients had relatively equal numbers of CD34^+^ and CD34^−^ LKS cells (Figure 12c, right). Sixteen weeks after tertiary transplant, total peripheral blood leukocytes decreased five fold with an even more significant reduction in the frequency of B cells (Figure 12d). Additionally, there was a striking reduction and, in some KO FL recipients, a complete absence, of CD45.2^+^ LKS cells, CD34^−^ LKS cells, and LKS CD34^−^ SLAM long term HSC at this time point (Figure 12e). These serial transplantation experiments suggest that the absence of Tle4 leads to reduced self-renewal of HSC.

## Discussion

In Drosophila, *Groucho* is a master transcriptional regulator expressed ubiquitously throughout development and influences multiple key developmental processes [7]. The precise roles of the various TLE homologues in vertebrates are not very well understood. To characterize those functions specific to *Tle4*, we developed a novel *Tle4* null mouse model. Our studies have identified the critical importance of *Tle4* in bone calcification, bone marrow niche formation, BM cellularity, B cell development, HSPC self-renewal capacity, and thymic and splenic architecture.

One of the striking abnormalities in these KO mice is a decreased calcification of the skeleton. This impaired ossification is apparent in both membranous bone (flat skull bones) as well as endochondral bones (long bones) by birth. The phenotype of impaired mineralization and abnormal growth plates in *Tle4* null mice is similar to that reported in mice lacking *Grg/Aes*, a truncated member of the Groucho/TLE gene family [20]. However, the defects in *Tle4* null mice are more profound and worsen with age instead of phenotypic recovery seen in *Grg5* null mice. Given the reported function of *Grg5* as a dominant negative inhibitor of Tle proteins, it might be surprising to see a similar phenotype between loss of *Grg5* and *Tle4*, however, this dominant negative effect is only demonstrated in some over-expression or mis-expression studies and evidence points to other roles for *Grg5* independent of its ability to inhibit the Tles, and in some contexts has similar repressive functions as the Tles [21]. The Groucho/TLE proteins are capable of interacting with multiple signaling pathways and may alter the function of key proteins important for bone formation, including Runx2/Cbfa1, a critical regulator of bone development and maturation [14], [22]. The apparent resorption of trabeculae seen in 4 week old mice under the growth plate suggests additional abnormalities in addition to bone formation. Further investigations are underway to better characterize the nature of this defect in bone development and maintenance.

Several reports have described the role of the BM microenvironment on hematopoiesis [23], [24]. Osteoblasts have a well-defined role in supporting B lymphopoiesis via expression of the heterotrimeric G protein alpha subunit G(s)alpha [25], [26]. While we have not yet determined whether osteoblasts are specifically affected in our model *per se*, it is reasonable to assume that the compromise of trabecular bone is at least contributory to the observed B cell and HSPC defects. The inability of *Tle4* null stromal cells cultured *in* *vitro* to maintain WT HSC suggests that the hematopoietic phenotype seen in KO mice may derive in part from niche-induced deregulation. Furthermore, as evidenced by TUNEL staining of bones harvested from WT and KO mice, it is clear that the absence of *Tle4* has an effect on the viability and integrity of the BM niche and stroma, Further experiments are needed to better characterize the nature of this defective stromal support of HSPC.

Loss of *Tle4* appears to significantly impair LSK differentiation into granulocyte, monocyte, macrophage progenitors and LSK self-renewal, at least in part due to increased cellular apoptosis. The finding of preserved numbers of long term stem cells as marked by CD34+ LSK CD48− CD150+ in two week old *Tle4* knockout mice, despite decreases in more mature lineages, further studies are need to understand the mechanisms of this preservation.

Concurrently, BM and fetal liver serial transplantation experiments demonstrate a robust HSPC-intrinsic effect of *Tle4* deletion. In both transplant models, mice receiving KO HSPC develop peripheral leukopenia. Moreover, this finding in FL serial transplantation illustrates the potential HSPC-intrinsic defects of Tle4 loss leading to decreased capacity of HSPC self-renewal. Additionally, our study provides the first direct *in* *vivo* evidence of a role of Tle4 on B-cell development, an effect previously inferred based on interactions of Tle4 and Pax5 [28],[29]. The somewhat distinct block in B-cell differentiation seen with Tle4 loss compared to that reported with Pax5 loss suggests Tle4 may exert some B-cell effects independent from Pax5, although we can’t exclude potential animal models differences as accounting for this effect. Taken together, our data demonstrates the critical importance of *Tle4* in regulating various developmental processes central to bone maturation, medullary hematopoiesis, and HSPC maintenance. These findings may have significant implications for understanding hematopoiesis in both normal and disease states. Moreover, our observations provide further insight and affirmation to previous findings that implicate *Tle4* as a critical regulator of leukemia and other states of hematological dysregulation.

## Conclusion

In summary, by the development of the first model for *Tle4* deletion in mammals, our data provide evidence for an essential role for *Tle4* in mammalian bone and blood development. *Tle4* deficient B cells exhibit intrinsic developmental defects and HSC exhibit stem cell depletion after serial transplantation. *Tle4* null bone marrow stromal cells fail to support hematopoiesis *in* *vitro*, suggesting a potentially novel extrinsic role of *Tle4* in the regulation of hematopoiesis. *Tle4* null mice have profound defects in bone mineralization and growth plate organization, which apparently affects skeletal growth.

Our work has shed light on a novel regulatory function for this corepressor in normal hematopoiesis and bone development. As such, elucidating the regulatory mechanisms controlled by *Tle4* offers a significant challenge and opportunity for expanding our understanding of bone development and the multicellular orchestration of hematopoiesis. This work further potentially offers novel insight into the transcriptional processes underlying malignant transformation.
